# LncRNA–protein interaction prediction with reweighted feature selection

**DOI:** 10.1186/s12859-023-05536-1

**Published:** 2023-10-30

**Authors:** Guohao Lv, Yingchun Xia, Zhao Qi, Zihao Zhao, Lianggui Tang, Cheng Chen, Shuai Yang, Qingyong Wang, Lichuan Gu

**Affiliations:** School of Information and Computer, Anhui Agricultural University, Hefei, 230036 Anhui China

**Keywords:** LncRNA–protein prediction, Protein sequence, Feature selection, Boosting, Reweighting

## Abstract

LncRNA–protein interactions are ubiquitous in organisms and play a crucial role in a variety of biological processes and complex diseases. Many computational methods have been reported for lncRNA–protein interaction prediction. However, the experimental techniques to detect lncRNA–protein interactions are laborious and time-consuming. Therefore, to address this challenge, this paper proposes a reweighting boosting feature selection (RBFS) method model to select key features. Specially, a reweighted apporach can adjust the contribution of each observational samples to learning model fitting; let higher weights are given more influence samples than those with lower weights. Feature selection with boosting can efficiently rank to iterate over important features to obtain the optimal feature subset. Besides, in the experiments, the RBFS method is applied to the prediction of lncRNA–protein interactions. The experimental results demonstrate that our method achieves higher accuracy and less redundancy with fewer features.

## Introduction

Long non-coding RNAS, also known as lncRNAs, is a series of single-stranded polynucleotides (no less than 200 nucleotides each), consisting of non-protein coding transcripts [1]. lncRNAs play a key role in various biological processes such as gene expression regulation, epigenetic regulation, and cell differentiation, attracting widespread attention in recent years [2]. LncRNAs can interact with proteins, DNA, and other RNA molecules. Among them, lncRNA–protein interactions (LPIs) have been widely studied due to their key roles in cellular processes and contributions to understanding the molecular mechanisms of various diseases, including cancer, neurodegenerative diseases, and cardiovascular diseases.

Originally, biologists detected lncRNA–protein interactions by biology experiments, such as RNA pulldown [3], Crosslinking and Immunization (CLIP) [4], Capture Hybridization Analysis of RNA targets (CHART) [5] and RNA Binding Protein Immunoprecipitation (RIP) [6]. Although these methods can distinguish lncRNA–protein interactions reliably, they are too cumbersome and time-consuming for high throughput screening of lncRNA–protein interactions. The use of computational intelligence in identifying LncRNA–protein Interactions (LPI) has attracted significant attention from researchers due to its potential benefits, including cost reduction in laboratory experiments and improved speed and accuracy [7].Therefore, many prediction algorithms based on machine learning have been developed to predict LPI.

Machine learning techniques can predict LPIs by integrating various features, such as sequence, structure, and functional information. For example, lncpro [8] is developed to extract the secondary structure of RNA and protein, which can effectively discriminate between interacting and non-interacting RNA–protein pairs and predict RNA–protein interactions within a given complex. SFPEL-LPI [9] is an extracted method of the feature vector of lncRNA and protein, it define the interaction spectrum between lncRNA and protein according to the known lncRNA–protein interaction. learning framework to make predictions. Shen et al. proposed LPI-KTASLP to identify lncRNA–protein interactions with kernel target alignment and a semi-supervised link prediction model using multivariate information [10]. DeepLPI [11] is a multimodal deep learning method for predicting the interactions between lncRNAs and protein isoforms, which can predict the interactions between lncRNAs and protein isoforms with corresponding confidence scores. Besides, during the training of a machine learning model, the presence of noise or irrelevant information in the input data can lead to inconsistencies or misleading patterns that the model may mistakenly learn. Consequently, this can result in a decrease in the accuracy of the model. To mitigate the negative impact of data noise, it is important to preprocess the data by removing outliers, cleaning up errors, and filtering out irrelevant features. Reweighting in machine learning refers to the adjustment of the importance or contribution of training instances or features in a learning algorithm [12]. It can address the issue of noisy data or give more emphasis to certain examples or features during the learning process. The reweighting model can focus on the important or minority instances, leading to better performance and results. Besides, the high dimensionality of the feature space and the complexity of LPI on performance of the prediction model are key problems predicting LPI.

Feature selection is a key step in building an accurate LPI model, which can reduce the dimension of the feature space, reduce overfitting, and improve the generalization ability of the model [13]. The common feature selection methods applied in LPI include: Filter method, it evaluates the statistical properties of each feature independently of the target variable, such correlation analysis, chi-square tests, and mutual information [14]. Wrapper methods, it assess the quality of features by considering their impact on the performance of a specific machine learning algorithm [15]. The embedded method, incorporates feature selection as part of the model building process itself. Techniques like LASSO (Least Absolute Shrinkage and Selection Operator) and Ridge regression apply regularization to penalize irrelevant features, effectively performing feature selection [16]. Therefore, these feature selection methods can be effectively utilized to mine lncRNA features related to protein interactions and improve prediction accuracy while reducing the computational cost.

In conclusion, our study presents a novel approach, reweighting boosting feature selection (RBFS), for addressing the challenge of lncRNA–protein interaction (LPI) prediction. We construct an extensive feature set by integrating diverse bioinformatics data sources, and leveraging sample reweighting to select optimal feature sets for LPI prediction. Through multiple iterations of feature ranking using feature selection, we obtain a robust and accurate final LPI prediction model based on the best feature set.

Extensive evaluations on multiple benchmark datasets demonstrate the effectiveness of RBFS. Comparative analysis against four state-of-the-art LPI prediction methods reveals that RBFS outperforms them in terms of key performance metrics including recall, precision and F1 score, as well as the area under the receiver operating characteristic curve (AUROC) and the area under the precision-recall curve (AUPR).

Furthermore, we conduct a comprehensive analysis of the selected features, providing valuable insights into the underlying molecular mechanisms of LPIs and their potential implications in disease pathogenesis. Our findings contribute to the advancement of LPI prediction and offer promising avenues for further research in this field. The main contributions of this paper are summarized as follows:

1. Reweighting can decreases the importance of outliers or noisy data to improve the performance of model.
2. Feature selection can identify and select relevant important features.
3. The results of testing indicate that the RBFS method has better performance than other existing methods.

The rest of the paper is organized as follows: In “Related work” section, this paper mainly introduces some work related to survival risk prediction. In “Methods” section, we describe the overall framework and specific content of the proposed method. In “Experimental design” section, the datasets, experimental setup and results of our work are elaborated and analyzed. Section “Conclusion” section provides a conclusion.

## Related work

### Protein sequence data representation method

Protein structure prediction and function recognition are critical tasks in proteomics research, which rely on the analysis and research of protein data information [17]. Several statistical analysis methods, including support vector machines, genetic algorithm Bayesian networks, decision trees, and hidden Markov models, have been widely used for protein data analysis. To produce reliable and true results, correctly extracting protein sequence information features is crucial in the early stages. Protein sequence information feature extraction can be divided into three categories: (1) amino acid composition-based methods, (2) physicochemical properties of amino acid residues-based methods, and (3) combination information-based methods. Sequence-based feature extraction methods have been widely applied, and the choice of feature extraction methods varies depending on the context of the application [18].

**Fig. 1:**
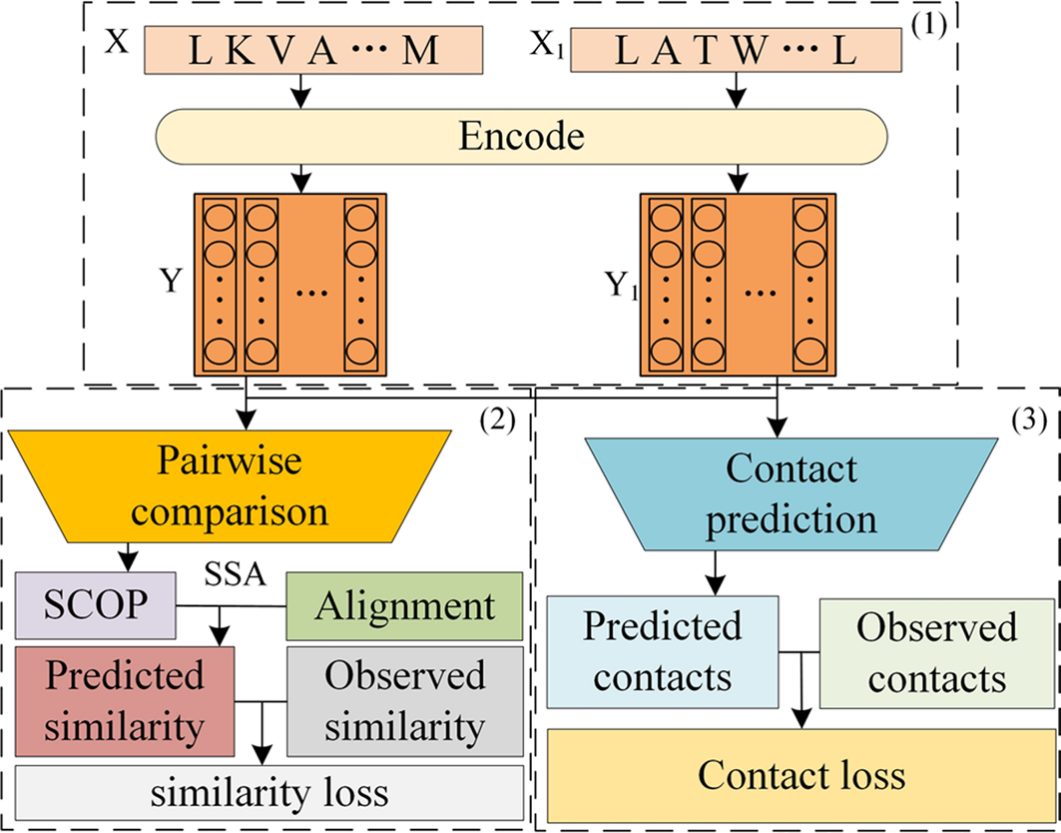
Diagram of the model for feature extraction from sequence information. (1) The encoder model transforms amino acid sequences into sequences of vector embeddings. (2) The similarity prediction module utilizes pairs of proteins represented by their vector embedding sequences to predict their shared structural classification of proteins (SCOP) level. Sequence alignment is performed based on the L1 distance between their vector embeddings, employing the sequence-structure alignment (SSA)method. Subsequently, a similarity score is computed from the alignment and linked to shared SCOP levels through ordinal regression. (3) The contact prediction module leverages the vector embedding sequence to predict contacts between amino acid positions within each protein. The contact loss is determined by comparing these predictions with observed contacts in the protein’s 3D structure. The parameters of the encoder are adjusted by utilizing error signals from both tasks

In recent years, deep learning techniques have gained significant attention for their application in representing protein sequence features. Notably, Bepler et al. introduced a model that effectively maps protein sequence information to embedded vectors, as depicted in Fig. 1. In this model, each amino acid is assigned a specific encoding that captures structural information, thereby facilitating comprehensive feature representation of protein sequences [19]. The model leverages a bidirectional long short-term memory (BiLSTM) [19] architecture to learn the global structural similarity between protein networks and the characteristics of individual proteins based on their sequences. Furthermore, this approach extends to extracting features from RNA sequences. Specifically, for protein and RNA sequences, the BiLSTM model takes each amino acid or nucleotide as input and processes them through two LSTM layers, one in the forward direction and the other in the backward direction. This allows for considering both the information at the current position in the sequence and the information from its surrounding positions. By learning patterns and features within the sequence, the BiLSTM model can transform protein and RNA sequences into continuous vector representations for subsequent prediction tasks.

Additionally, to incorporate local structural context within proteins, the framework includes position-level supervision derived from residue-residue contacts within a single protein structure. The model effectively utilizes both the overall structural similarity between proteins and the residue-residue contacts within individual proteins for model training.

### LncRNA–protein interaction prediction methods

Predicting interactions between long non-coding RNAs (lncRNAs) and proteins has been a significant research focus in recent years due to its critical role in various biological processes, such as gene regulation, cell differentiation, and disease development. Several computational methods have been proposed to predict lncRNA–protein interactions, including sequence-based, structure-based, and network-based methods. Sequence-based methods use sequence information, such as k-mer frequency and sequence composition, to represent lncRNAs and proteins. Structure-based methods rely on the 3D structures of lncRNAs and proteins to infer their interactions. Network-based methods leverage the topological properties of biological networks to predict lncRNA–protein interactions [20–22].

### Feature selection techniques in lncRNA–protein interaction prediction

Feature selection plays a crucial role in improving prediction performance by reducing the dimensionality of the feature space and mitigating the risk of overfitting. In the prediction of lncRNA–protein interactions, several feature selection techniques have been employed, including filter methods, wrapper methods, and embedded methods. The RBFS (Relevance-Based Feature Selection) method is an innovative approach that combines the advantages of filter and wrapper methods. By iteratively adjusting the importance weights of features based on their contributions to prediction performance, this method selects the most relevant features while minimizing the risk of overfitting. Promising results have been demonstrated by applying this approach to various bioinformatics tasks such as gene expression data analysis and protein function prediction [23, 24].Applying the RBFS method to the prediction of lncRNA–protein interactions can significantly improve prediction performance and contribute to a deeper understanding of lncRNA–protein interactions in biological systems.

However, in feature selection, we also face another important challenge, which is how to avoid performance estimation bias due to information leakage. Particularly in lncRNA–protein interaction prediction, certain lncRNAs may interact with multiple proteins, which can lead to information leakage issues in cross-validation [25, 26]. To address this problem, we plan to construct a new dataset where each lncRNA–protein pair represents a unique interaction [27]. By ensuring that there is no overlap of interaction pairs between the training and testing sets, we can avoid the issue of information leakage and more accurately evaluate the performance of feature selection techniques. By adopting this approach, we can obtain more reliable assessments of feature selection techniques’ performance in lncRNA–protein interaction prediction while reducing potential bias resulting from information leakage. Furthermore, this methodology has the potential to significantly enhance prediction performance and contribute to a deeper understanding of lncRNA–protein interactions in biological systems.

## Methods

**Fig. 2:**
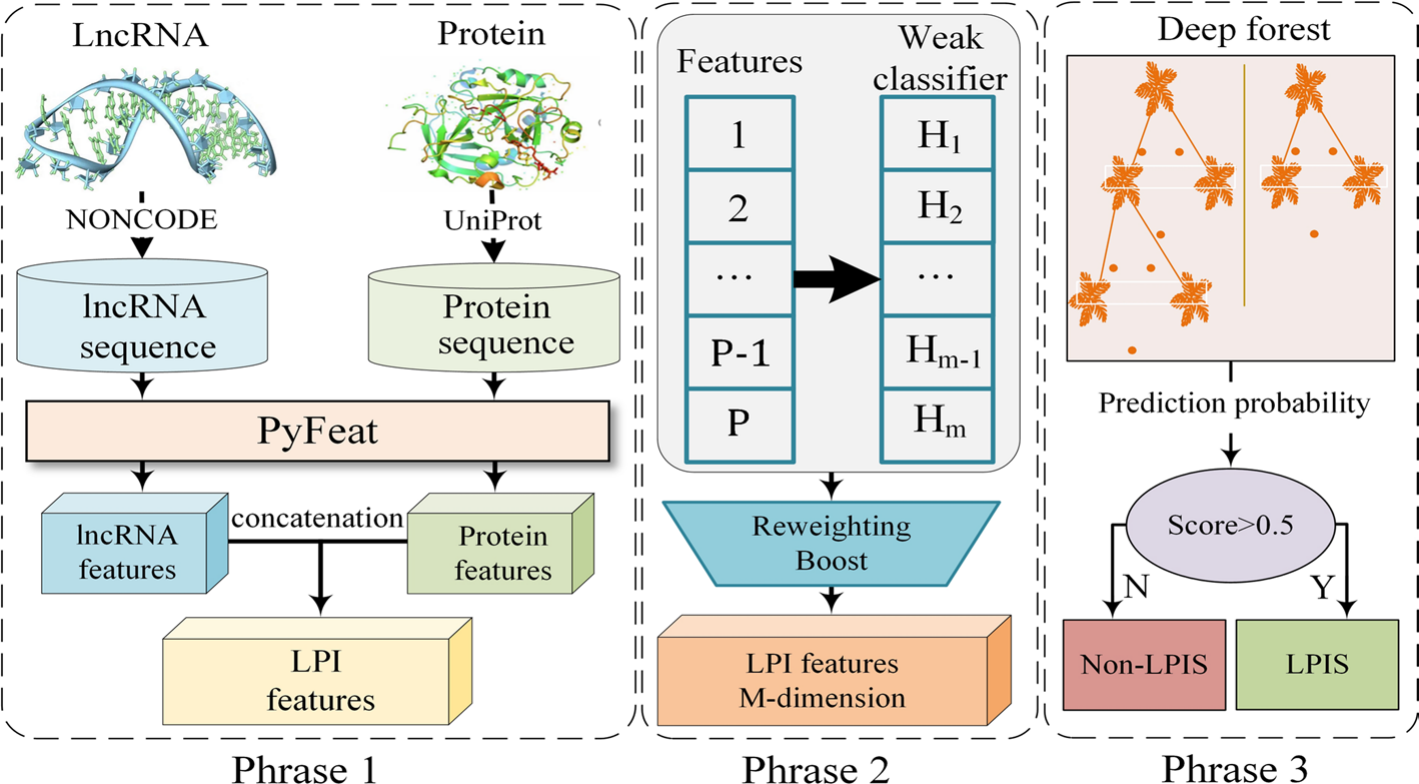
The workflow of the RBFS framework is as follows: (1) Initial feature acquisition. lncRNA and protein features [28] are obtained using Pyfeat [29] and concatenated to represent each lncRNA–protein pair. (2) Feature selection. The concatenated features are subjected to dimensionality reduction using Reweighting Boost. (3) LPI classification. XGBoost is designed to capture unobserved LPIs

The Reweighing-Boost method is based on the feature ranking obtained from XGBoost and iteratively determines the optimal feature set. In this study, the Reweighing-Boost method is applied to predict long non-coding RNA–protein interactions. The experimental results demonstrate that our method outperforms other existing methods in terms of recall, precision, F1 score, and other metrics. Moreover, our method also achieves superior performance in terms of the area under the receiver operating characteristic curve (AUROC) and the area under the precision-recall curve (AUPR) compared to other existing methods. Furthermore, RBFS achieves a reduced and non-redundant feature set. Figure 2 illustrates the workflow of our method. High-dimensional features can contain comprehensive information, but they also require significant time for data training and model configuration. In order to reduce the dimensionality of the data, feature selection methods are employed in this study to extract effective features from protein and RNA sequence data, eliminating redundant features and reducing the impact of irrelevant features on classification performance. Boosting algorithms are commonly used ensemble classification meta-algorithms that can be utilized to modify the feature search space in wrapper-style feature selection methods. This paper proposes a method called Reweighting Boosting Feature Selection (RBFS), which utilizes feature importance scores embedded in the ensemble tree model to select candidate features. The Boosting algorithm is then employed to update the feature importance scores after each iteration, thereby updating the search space.

Compared with the results of previous studies, this paper proposes the following methods: (1) a sample weighting strategy, which updates the weight value of the sample according to the prediction results of the previous prediction model. In contrast, the previous method used the same weights for all classified samples. (2) A modular algorithm structure is adopted to decouple feature ranking and feature selection. This overcomes the inconsistency in feature ranking and potentially increases the robustness of the selected subset. AdaBoost’s weighting strategy is to add the same weight value to all misclassified samples [30], which ignores the gap between the sample and its correct class. Therefore, the weighting strategy assumes that the weight of each sample is inversely proportional to its prediction probability. The specific formula is shown in Eq. 1:

1 $$\begin{aligned} & \partial ^i=-\sum _{c=1}^{n_i} Y_c \log \left( P_c\right) \end{aligned}$$

2 $$\begin{aligned} & \partial _j^i=\partial _j^i / \partial _j^{i-1}; \forall j=1, \ldots , n \end{aligned}$$

3 $$\begin{aligned} & \omega _j^{i+1} \leftarrow \omega _j^i \cdot \partial _j^i; \forall j=1, \ldots , n \end{aligned}$$

4 $$\begin{aligned} & \omega _j^{i+1}=\frac{\omega _j^{i+1}}{\sum _{j=1}^n \omega _j^{i+1}}; \forall j=1, \ldots , n \end{aligned}$$

In Eq. 1, the one-hot encoding matrix represents the correct class of each sample and is obtained by a classifier, which contains an n*n matrix of probabilities for each sample. Equations 3 and 4 represent the weight values of sample j in the ith iteration. For a sample, when its correct probability is close to 1, the weight decreases, and correspondingly, when its correct probability is close to 0, the weight term increases. Based on this principle, the model assigns higher weights to samples that are far from being classified correctly in this iteration.

In this study, the gradient boosting tree model XGBoost proposed by Chen [31] was used as the initial score getter for all samples. Although XGBoost has higher computational requirements compared to a single tree model, it is more accurate in the 6000+ dimensional sequence data used in this study, and its feature scoring reflects the complex interactions between lncRNA and proteins better. Many scholars [32–34] have used XGBoost for model training. The model used in this study consists of two parts. Firstly, XGBoost is used to obtain the initial scores for all features, generate the ranking of all features based on the tree-based classifier, and evaluate the performance of the top q features using the classifier. In k-fold cross-validation, the q features are sequentially added to the selected feature set, and the classification results of the model are evaluated. The best-performing feature is selected and added to the selected feature set. Then, for the features in the feature subset, the classifier is trained using all selected features, and the sample weights for subsequent iterations are updated using their predicted probabilities according to Eq. 1. After multiple iterations, a sample with p features is reduced to m features.

The algorithm sets a reset strategy for sample weights. Specifically, when the ith iteration is performed, if a feature that is already in the set is selected or the selected feature does not improve the classifier’s classification performance in this round of iteration, the algorithm temporarily terminates and uses the current feature set as the final feature set, resetting the sample weights to 1/*n*. The iteration is repeated i times until the termination condition occurs again. That is, the termination condition of the algorithm is that a feature is selected twice or the selected feature in a certain iteration does not improve the classification accuracy of the classifier.

## Experimental design

### Datasets

Five data sets are used in this paper, each of which contains protein sequence information, lncRNA sequence information and LPI network. Datasets human 1- human 3 are obtained from Li [35], Zheng [36] and Zhang et al. [37], respectively, and the three obtained data sets are preprocessed. UniProt [38], NPInter [39], NONCODE [40] and SUPERFAMILY [41] interaction data with only one relevant lncRNA or protein interaction and no sequence or protein expression information were removed. Datasets of Arabidopsis and maize are also obtained from Bai as dataset Arabidopsis and dataset maize of this paper, and their protein sequences, lncRNA sequences and LPI data are obtained from PlncRNADB [42]. The details of the data sets are shown in Table 1. In this paper, each LPI is defined as a matrix Y, as shown in Eq. 5.

5 $$\begin{aligned} y_{i j}=\left\{ \begin{array}{l} 1, \; \text{ Interaction } \text{ exists } \\ 0, \; \text{ No } \text{ Interaction } \text{ exists } \end{array}\right. \end{aligned}$$

These three data sets are human data, in which data set human 1 downloads the known lncrna–protein interaction data set in November 2013 from NPInter2.0, and selects the biological restriction of lncrna as “Homo sapiens”, the type restriction as “NONCODE”. The lncrna–protein interaction data set was screened. Then, according to the human lncrna data set in the NONCODE4.0 database, the above lncrna data were screened to obtain the results, and the lncrna ID and protein ID were mapped to the NONCODE4.0 ID and string ID. Datasets human 2 and human 3 were processed similarly to human 1, except that the data were downloaded from NPInter in different years, and new lncrna and protein interactions were discovered and updated to the database as the research progressed.

**Table 1:** Dataset presentation

Dataset	lncRNA	Protein	LPI pair	Non-LPI pair
Human 1	935	59	3479	51686
Human 2	885	84	3265	65536
Human 3	990	27	4158	22572
Arabidopsis	109	35	948	948
Maize	1704	42	22133	22133

### Evaluation metrics

In order to prove the learning performance of the RBFS method, a k-fold crossover experiment is conducted for the experimental results, with K = 5. Precision, Recall, Precision, F1-score, AUC value and AUPR value are used to evaluate the performance of RBFS for the prediction results of LPI. Each metric is defined as follows:

6 $$\begin{aligned} & \text{ Precision }=\frac{T P}{T P+F P} \end{aligned}$$

7 $$\begin{aligned} & \text{ Recall }=\frac{T P}{T P+F N} \end{aligned}$$

8 $$\begin{aligned} & \text{ Accuracy }=\frac{T P+T N}{T P+F P+T N+F N} \end{aligned}$$

9 $$\begin{aligned} & F1{-}score = \frac{2 \times {Precision} \times {Recall}}{{Precision}+{Recall}} \end{aligned}$$

In the above, TP, FP, TN and FN represent the predicted values of true LPIs, false LPIs, true non-LPIs and false non-LPIs respectively, and AUC and AUPR represent the average areas of ROC curve and precision-recall curve respectively. Considering that there is some noisy data in the high-throughput experimental data, this paper also proves the stability of the model through noise experiments. By adding noise data to five data sets, it proves that the proposed model has certain stability and robustness.

### Feature selection result analysis

In this section, RBFS is compared to five popular algorithms: LPI-HyADBS, LPI-SKF, LPI-NRLMF, and LPI-ETSLP. LPI-HyADBS is a feature selection framework that combines AdaBoost, deep neural network (DNN), XGBoost, and support vector machine (SVM) with penalty misclassification coefficient (C-SVM) [43]. LPI-SKF is a prediction model that integrates multiple similarities of lncRNAs and proteins using the SKF method to obtain a comprehensive similarity matrix. The LapRLS framework is then applied to build the prediction model [44]. LPI-NRLMF is a matrix factorization computational approach that utilizes a semi-supervised method without the need for negative samples. It integrates multiple similarities of lncRNAs and proteins and constructs a prediction model using neighborhood regularized logistic matrix factorization [45]. LPI-ETSLP is a semi-supervised link prediction method based on eigenvalue transformation, aiming to uncover the relationships between lncRNAs and proteins [46]. In this study, we employed a 5-fold cross-validation approach to evaluate the performance of the proposed model and to better demonstrate the experimental results. Specifically, we divided the random lncRNA–protein pairs in the LPI matrix Y into 5 subsets, with one subset serving as the test set while the remaining 4 subsets were used as the training set in a rotating fashion. Additionally, we reserved an independent validation set that was not used throughout the entire research process. This validation set was used for further validation of the selected model. By conducting multiple rounds of cross-validation and evaluating the model on the independent validation set, we aimed to mitigate the issue of similarity between the training and test sets and reduce the risk of overfitting.

**Table 2:** Contrast to existing methods across datasets

Data set	Method	Precision	Recall	Accuracy	F1
Human 1	LPI-HyADBS	0.699	0.344	0.619	0.475
	LPI-SKF	0.600	0.400	0.567	0.480
	LPI-NRLMF	0.459	0.369	0.467	0.409
	LPI-ETSLP	0.452	0.513	0.445	0.481
	RBFS	0.712	0.804	0.8	0.747
Human 2	LPI-HyADBS	0.754	0.704	0.77	0.754
	LPI-SKF	0.512	0.500	0.511	0.5057
	LPI-NRLMF	0.534	0.561	0.536	0.547
	LPI-ETSLP	0.4524	0.5135	0.4459	0.481
	RBFS	0.812	0.898	0.87	0.818
Human 3	LPI-HyADBS	0.553	0.609	0.559	0.58
	LPI-SKF	0.555	0.531	0.553	0.543
	LPI-NRLMF	0.533	0.307	0.519	0.390
	LPI-ETSLP	0.571	0.363	0.545	0.444
	RBFS	0.66	0.795	0.754	0.72
Arabidopsis	LPI-HyADBS	0.532	0.6	0.536	0.564
	LPI-SKF	0.611	0.282	0.551	0.386
	LPI-NRLMF	0.450	0.225	0.475	0.300
	LPI-ETSLP	0.517	0.428	0.514	0.468
	RBFS	0.714	0.687	0.763	0.699
Maize	LPI-HyADBS	0.703	0.77	0.729	0.74
	LPI-SKF	0.5714	0.3636	0.545	0.444
	LPI-NRLMF	0.500	0.378	0.500	0.430
	LPI-ETSLP	0.533	0.307	0.519	0.390
	RBFS	0.712	0.71	0.758	0.747

**Fig. 3:**
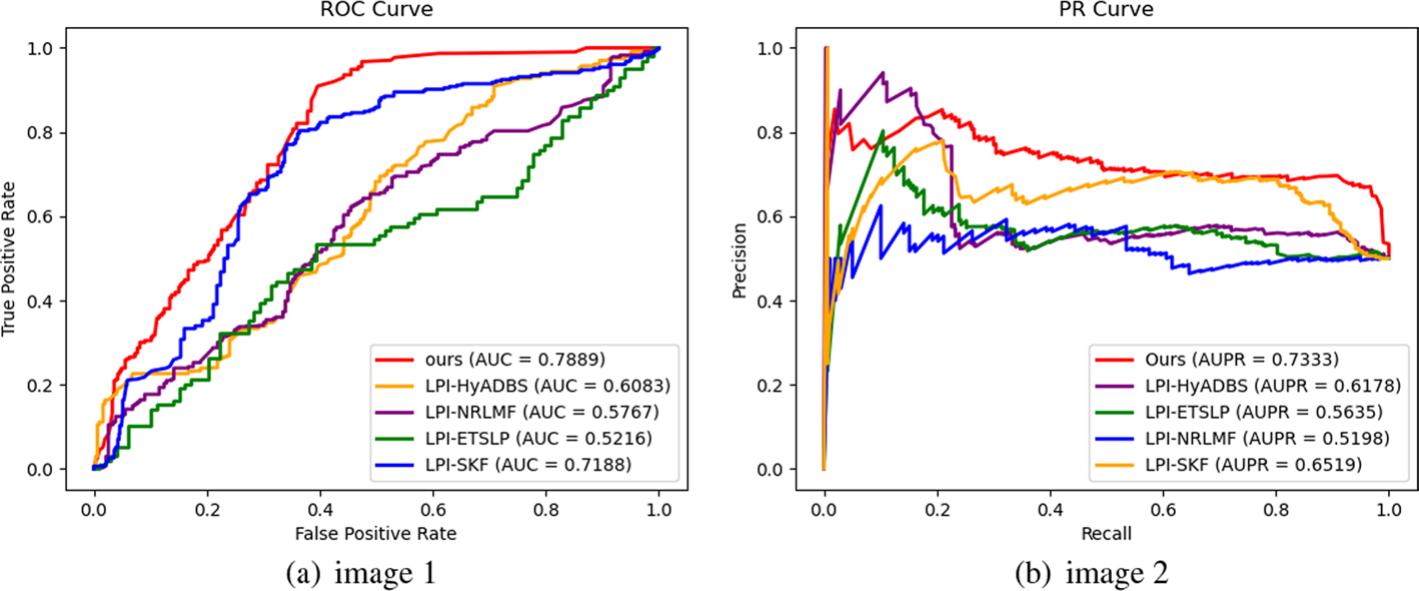
Indicator contrasts on dataset human 1

**Fig. 4:**
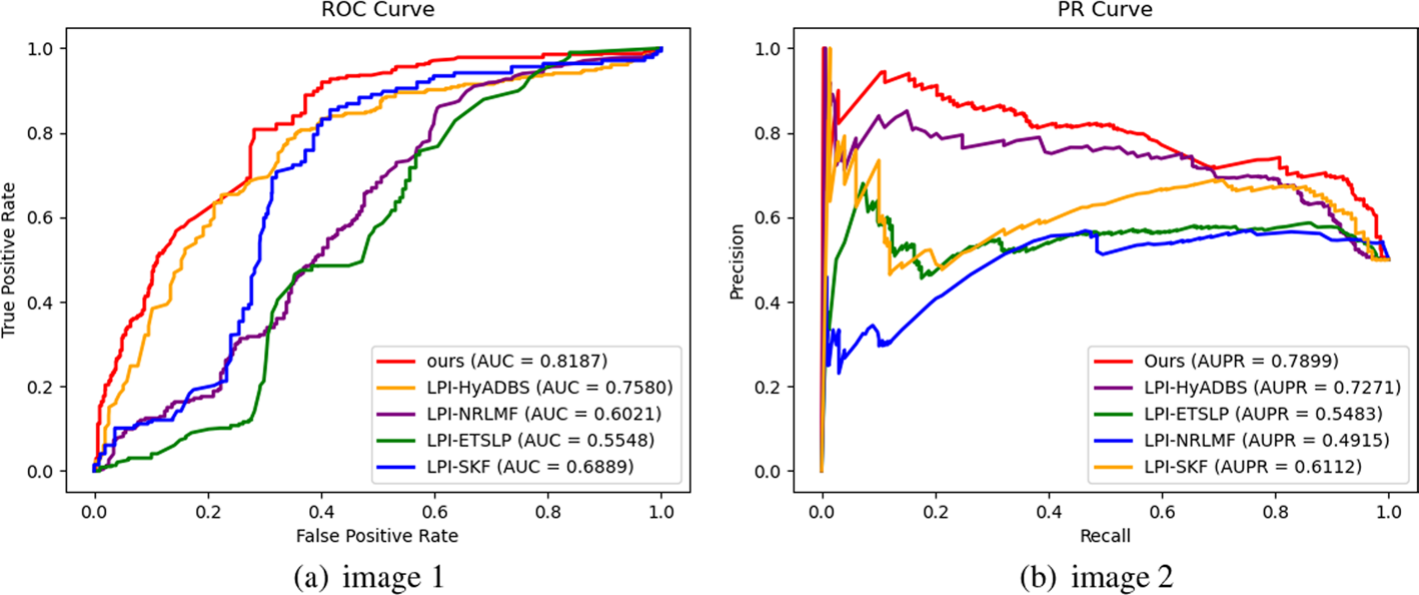
Indicator contrasts on dataset human 2

**Fig. 5:**
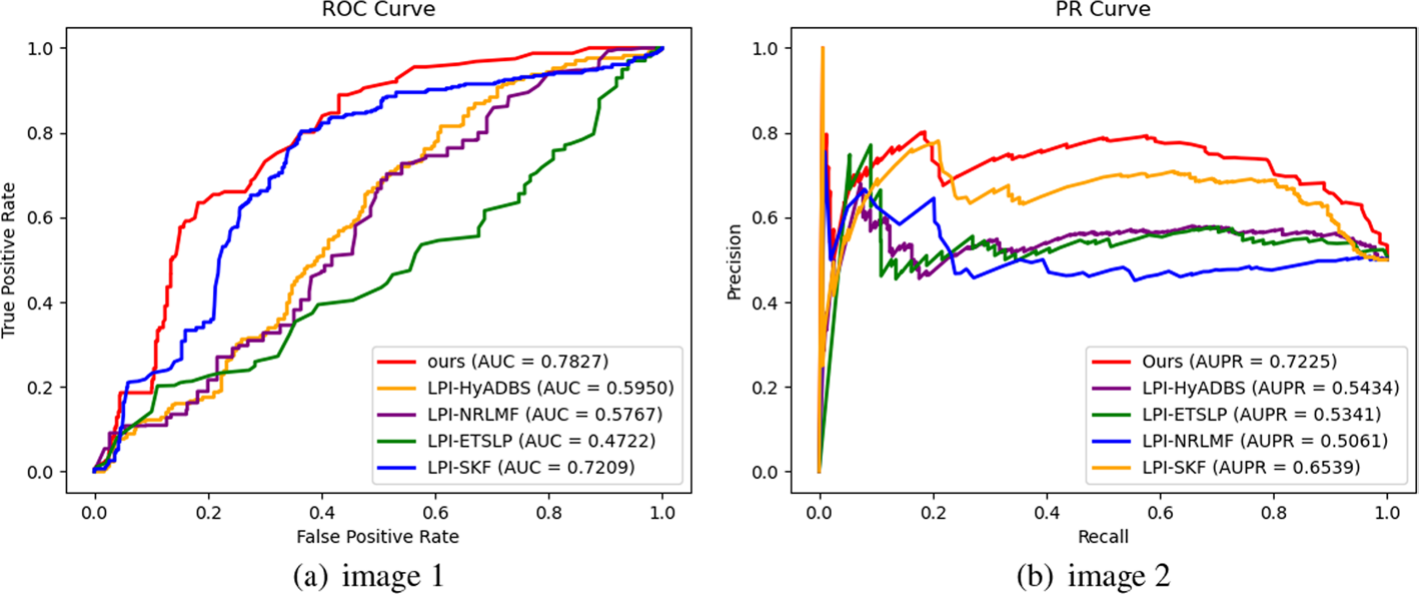
Indicator contrasts on dataset human 3

**Fig. 6:**
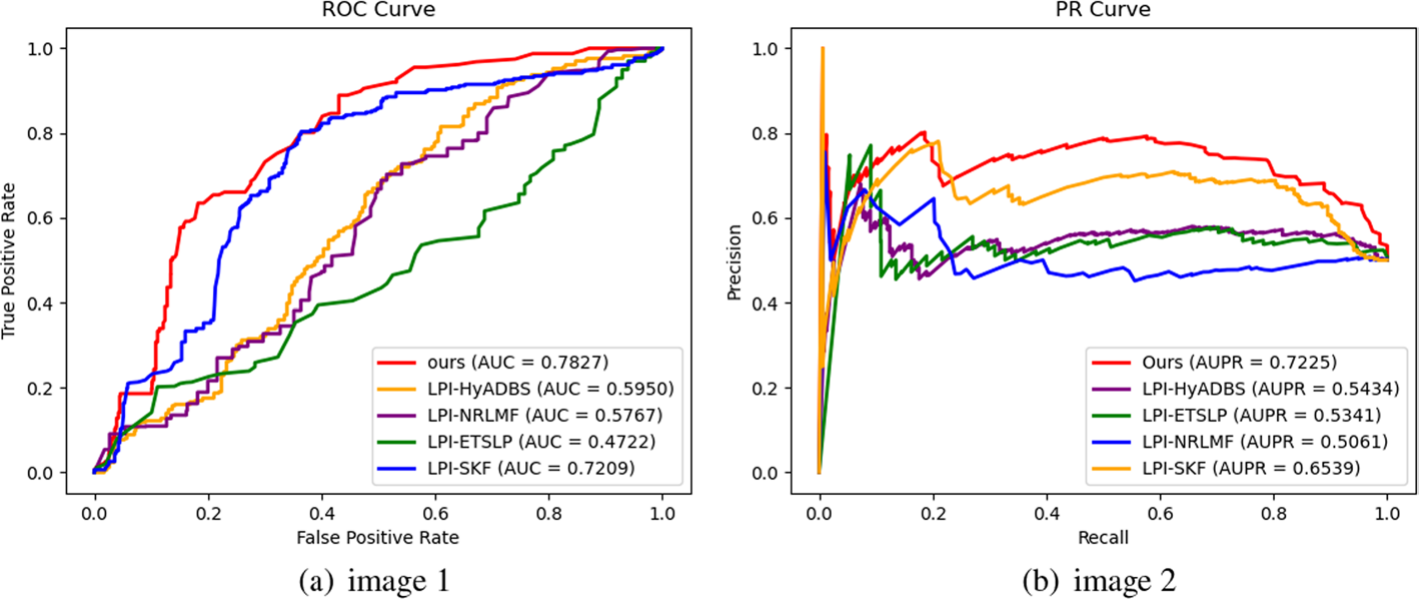
Indicator contrasts on dataset Arabidopsis

**Fig. 7:**
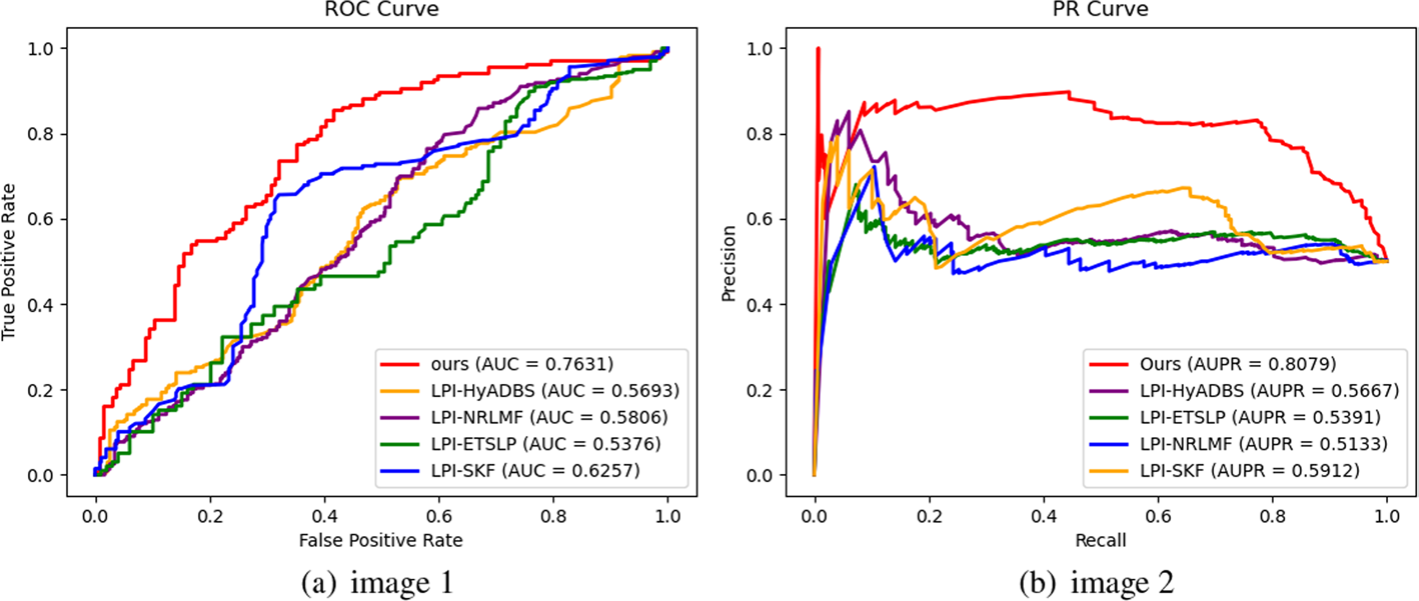
Indicator contrasts on dataset maize

Table 2 presents the Precision, Recall, F1-score, and Accuracy values of RBFS, LPI-HyADBS, LPI-SKF, LPI-NRLMF, and LPI-ETSLP during cross-validation on the five datasets. Figures 3, 4, 5, 6 and 7 display the AUC and AUPR values of RBFS compared to the four comparative algorithms across the five datasets. These figures demonstrate that RBFS outperforms the four comparative algorithms in terms of AUC and AUPR values, particularly on the three human datasets (human 1, human 2, and human 3). Despite some limitations in handling large datasets, RBFS exhibits good generalization ability and robustness, indicating its superior performance in feature selection and lncRNA–protein interaction prediction.

### Model robustness validation

In order to further substantiate the advantages of the RBFS method in the field of feature selection, this study incorporated five sets of noise experiments conducted within each dataset. The specific procedure involved adding random noise data to the last row and last column of each dataset. The experimental results were then compared against four alternative methods. The experimental outcomes are presented in Tables 3, 4, 5, 6 and 7. Table 3 showcases the results of the RBFS method through its execution of five sets of noise experiments across the five datasets. From the detailed experimental results presented in Table 3, it becomes evident that the inclusion of noise data leads to an overall improvement in the experimental indicators compared to the original dataset. This finding signifies the robust stability of the RBFS model when confronted with noisy data. The datasets used in this study are derived from three different species, categorized into two distinct groups: animals and plants. Notably, the experimental indicators obtained through the RBFS method exhibit a high degree of consistency across the three species. Additionally, the analysis of the AUC and AUPR indicators on individual datasets reveals a relatively concentrated range of improvements resulting from the incorporation of noise data. The observed enhancement falls within a range of 0.1 for both indicators.

**Table 3:** Reweighting-boost noise experiment

	Group 1		Group 2		Group 3		Group 4		Group 5	
Dataset	AUC	AUPR	AUC	AUPR	AUC	AUPR	AUC	AUPR	AUC	AUPR
Human 1	0.864	0.853	0.832	0.827	0.824	0.816	0.837	0.821	0.845	0.849
Human 2	0.841	0.814	0.825	0.824	0.838	0.831	0.831	0.819	0.852	0.817
Human 3	0.731	0.726	0.716	0.715	0.729	0.720	0.735	0.727	0.731	0.718
Arabidopsis	0.774	0.763	0.764	0.746	0.771	0.762	0.768	0.775	0.752	0.763
Maize	0.777	0.764	0.773	0.754	0.784	0.769	0.766	0.778	0.743	0.768

**Table 4:** LPI-HyADBS noise experiment

	Group 1		Group 2		Group 3		Group 4		Group 5	
Dataset	AUC	AUPR	AUC	AUPR	AUC	AUPR	AUC	AUPR	AUC	AUPR
Human 1	0.769	0.738	0.574	0.728	0.658	0.719	0.769	0.734	0.658	0.739
Human 2	0.817	0.786	0.749	0.762	0.639	0.768	0.856	0.657	0.648	0.693
Human 3	0.514	0.500	0.498	0.499	0.507	0.527	0.566	0.449	0.502	0.476
Arabidopsis	0.890	0.878	0.777	0.867	0.758	0.839	0.807	0.644	0.839	0.633
Maize	0.873	0.856	0.836	0.863	0.794	0.777	0.804	0.880	0.819	0.861

**Table 5:** LPI-NRLMF noise experiment

	Group 1		Group 2		Group 3		Group 4		Group 5	
Dataset	AUC	AUPR	AUC	AUPR	AUC	AUPR	AUC	AUPR	AUC	AUPR
Human 1	0.696	0.654	0.602	0.688	0.643	0.701	0.698	0.632	0.598	0.564
Human 2	0.695	0.634	0.653	0.642	0.528	0.674	0.746	0.543	0.544	0.620
Human 3	0.493	0.486	0.488	0.412	0.453	0.465	0.501	0.421	0.499	0.465
Arabidopsis	0.688	0.668	0.564	0.659	0.549	0.627	0.601	0.439	0.640	0.432
Maize	0.646	0.646	0.626	0.673	0.694	0.547	0.614	0.610	0.619	0.681

**Table 6:** LPI-ETSLP noise experiment

	Group 1		Group 2		Group 3		Group 4		Group 5	
Dataset	AUC	AUPR	AUC	AUPR	AUC	AUPR	AUC	AUPR	AUC	AUPR
Human 1	0.589	0.538	0.374	0.458	0.458	0.619	0.569	0.574	0.518	0.569
Human 2	0.687	0.676	0.648	0.668	0.521	0.554	0.645	0.472	0.487	0.503
Human 3	0.345	0.356	0.320	0.345	0.422	0.415	0.482	0.488	0.355	0.465
Arabidopsis	0.743	0.715	0.684	0.701	0.681	0.723	0.689	0.531	0.721	0.423
Maize	0.656	0.645	0.623	0.684	0.589	0.572	0.591	0.576	0.657	0.663

**Table 7:** LPI-SKF noise experiment

	Group 1		Group 2		Group 3		Group 4		Group 5	
Dataset	AUC	AUPR	AUC	AUPR	AUC	AUPR	AUC	AUPR	AUC	AUPR
Human 1	0.754	0.723	0.572	0.756	0.678	0.754	0.776	0.754	0.687	0.762
Human 2	0.803	0.753	0.738	0.772	0.654	0.784	0.869	0.652	0.654	0.686
Human 3	0.525	0.513	0.500	0.504	0.511	0.528	0.567	0.460	0.509	0.465
Arabidopsis	0.856	0.868	0.772	0.843	0.755	0.842	0.809	0.654	0.840	0.634
Maize	0.869	0.852	0.843	0.878	0.805	0.784	0.801	0.879	0.834	0.841

Consequently, the RBFS method effectively demonstrates its prowess in feature selection across datasets originating from different species, thereby reinforcing its stability amidst noisy data. Tables 4, 5, 6 and 7 respectively present the outcomes of the four alternative methods in the context of five sets of noise experiments conducted across the five datasets. Overall, these methods exhibit substantial fluctuations in their experimental outcomes across the three species. Importantly, the experimental effects of these alternative methods diminish when compared to the original dataset subsequent to the inclusion of noise data. This trend highlights the limitations of these models in effectively dealing with noisy datasets. Moreover, analysis of the noise experimental outcomes on individual datasets reveals fluctuations within a range of approximately 0.2. In summary, the RBFS method model outperforms the aforementioned four alternative models in terms of its generalization ability and stability.

## Conclusion

In this paper, a five-fold cross-validation experiment was conducted on five datasets from three species, and the data sets after feature selection were applied to the prediction of lncRNA–protein interaction, which was evaluated with the existing methods on the evaluation index. The method proposed in this paper has a significant improvement in Precision, Recall, Accuracy and F1 score. In order to better illustrate the experimental effect of the RBFS method model on the imbalanced data set, the experimental results are compared with the existing lncrna–protein interaction prediction model LPI-HyADBS on the ROC curve and PR curve, and the results show that the proposed model has a better effect. Therefore, the RBFS method can effectively remove redundant information in the data set and predict lncrna–protein interactions by selecting an effective feature set.In addition, in order to illustrate the stability of the RBFS method on the data set and its generalization ability on lncrna–protein interactions of different species, this paper also sets up a noise experiment to explain the results of feature selection. To sum up, it can be concluded that the RBFS method model has better effects, better generalization ability and stability.

In future research, our attention will be directed towards several key areas. Firstly, we intend to validate the proposed model using larger lncRNA–protein datasets obtained from diverse data sources. This will further establish its effectiveness in real-world scenarios. Secondly, we aim to investigate alternative network structures to enhance the model’s performance even further. Additionally, we plan to refine the model architecture to effectively capture variable interactions. Lastly, we recognize the significance of applying lncRNA–protein analysis results in practical settings, particularly in patient prognosis management. This application has the potential to yield substantial practical benefits.

## Data Availability

The data and materials used in this study are not publicly available due to participant privacy concerns and the ongoing nature of the research. Interested researchers can contact the corresponding author for access to the code and data, subject to ethical and legal considerations.

## References

[1] Guttman M, Rinn JL (2012). Modular regulatory principles of large non-coding RNAs. Nature.

[2] Tiwari A, Srivastava R (2014). A survey of computational intelligence techniques in protein function prediction. Int. J. Proteomics.

[3] Batista PJ, Chang HY (2013). Long noncoding RNAs: cellular address codes in development and disease. Cell.

[4] Darnell RB. Clip (cross-linking and immunoprecipitation) identification of RNAs bound by a specific protein. Cold Spring Harbor Protoc. 2012;2012(11):pdb–prot072132. 10.1101/pdb.prot072132 23118367

[5] Simon MD, Wang CI, Kharchenko PV, West JA, Chapman BA, Alekseyenko AA, Borowsky ML, Kuroda MI, Kingston RE (2011). The genomic binding sites of a noncoding RNA. Proc Natl Acad Sci.

[6] Selth LA, Gilbert C, Svejstrup JQ. RNA immunoprecipitation to determine RNA–protein associations in vivo. Cold Spring Harbor Potoc. 2009;2009(6):pdb–prot5234. 10.1101/pdb.prot5234 20147192

[7] Jalali S, Kapoor S, Sivadas A, Bhartiya D, Scaria V (2015). Computational approaches towards understanding human long non-coding RNA biology. Bioinformatics.

[8] Lu Q, Ren S, Lu M, Zhang Y, Zhu D, Zhang X, Li T (2013). Computational prediction of associations between long non-coding RNAs and proteins. BMC Genomics.

[9] Zhang W, Yue X, Tang G, Wu W, Huang F, Zhang X (2018). SFPEL-LPI: sequence-based feature projection ensemble learning for predicting LncRNA–protein interactions. PLoS Comput Biol.

[10] Shen C, Ding Y, Tang J, Jiang L, Guo F (2019). LPI-KTASLP: prediction of LncRNA–protein interaction by semi-supervised link learning with multivariate information. IEEE Access.

[11] Shaw D, Chen H, Xie M, Jiang T (2021). DeepLPI: a multimodal deep learning method for predicting the interactions between lncRNAs and protein isoforms. BMC Bioinform.

[12] Zhou X, Lin Y, Pi R, Zhang W, Xu R, Cui P, Zhang T. Model agnostic sample reweighting for out-of-distribution learning. In: International conference on machine learning, PMLR; 2022. p. 27203–21.

[13] Alhenawi E, Al-Sayyed R, Hudaib A, Mirjalili S (2022). Feature selection methods on gene expression microarray data for cancer classification: a systematic review. Comput Biol Med.

[14] Wang W, Wang Y, Sun B, Liang S, Liu D, Zhang H, Wang X (2023). LPLSG: prediction of lncRNA–protein interaction based on local network structure. Curr Bioinform.

[15] Lihong P, Wang C, Tian X, Zhou L, Li K. Finding lncRNA–protein interactions based on deep learning with dual-net neural architecture. IEEE/ACM Trans Comput Biol Bioinform. 2021. 10.1109/TCBB.2021.3116232 34587091

[16] Ma Y, Zhang H, Jin C, Kang C (2023). Predicting lncRNA–protein interactions with bipartite graph embedding and deep graph neural networks. Front Genet.

[17] Zhao Z, Xu W, Chen A, Han Y, Xia S, Xiang C, Wang C, Jiao J, Wang H, Yuan X (2021). Protein functional module identification method combining topological features and gene expression data. BMC Genomics.

[18] Yang A. Research on feature extraction method and application of biological data, Ph.D. Thesis. Hunan University. 2012.

[19] Bepler T, Berger B. Learning protein sequence embeddings using information from structure. 2019. arXiv:1902.08661. PMC12947628 41768824

[20] Muppirala UK, Honavar VG (2011). RPI-Pred: predicting ncRNA–protein interaction using sequence and structural information. BMC Bioinform..

[21] Lu Q, Ren S-P, Lu M-J, Zhang Y-W, Zhu D-F, Zhang X-L, Li T, Liu Q-H, Zhang Y (2013). lncpro: an accurate and efficient predictor of protein-lncRNA interactions. Mol BioSyst.

[22] Yi H, Zhang L, Mou X, Xu Y, Cui Q, Zhang Y, Zhang Y, Zhang X (2020). Npinter v4.0: an integrated database of ncRNA interactions. Nucl Acids Res.

[23] Liu D, Zhang Y, Gao X, Xi J, Wang J, Feng X (2020). Feature selection in gene expression data analysis: a comprehensive review. Brief Bioinform.

[24] Xu J, Cai Y, Yu X, Zhu Y. Feature selection in protein function prediction: a review. Brief Bioinform. 2021.

[25] Whalen S, Truty RM, Pollard KS (2016). Enhancer-promoter interactions are encoded by complex genomic signatures on looping chromatin. Nat Genet.

[26] Cao F, Fullwood MJ (2019). Inflated performance measures in enhancer–promoter interaction–prediction methods. Nat Genet.

[27] Whalen S, Pollard KS (2019). Reply to ‘inflated performance measures in enhancer–promoter interaction–prediction methods’. Nat Genet.

[28] Xu W, Zhao Z, Zhang H, Hu M-J, Yang N, Wang H, Wang C, Jiao J, Gu L (2022). Deep neural learning based protein function prediction. Math Biosci Eng: MBE.

[29] Muhammod R, Ahmed S, Farid DM, Shatabda S, Sharma A, Dehzangi A (2019). PyFeat: a python-based effective feature generation tool for DNA, RNA and protein sequences. Bioinformatics.

[30] Freund Y, Schapire RE. A decision-theoretic generalization of on-line learning and an application to boosting. In: European conference on computational learning theory. 1997. https://api.semanticscholar.org/CorpusID:6644398.

[31] Chen T, Guestrin C. Xgboost: A scalable tree boosting system. In: Proceedings of the 22nd ACM SIGKDD international conference on knowledge discovery and data mining. 2016. https://api.semanticscholar.org/CorpusID:4650265.

[32] Luckner M, Topolski B, Mazurek M. Application of XGBoost algorithm in fingerprinting localisation task. In: Computer information systems and industrial management: 16th IFIP TC8 international conference, CISIM 2017, Bialystok, Poland, June 16–18, 2017, Proceedings. Berlin: Springer. 2017. p. 661–71.

[33] Alsahaf A, Azzopardi G, Ducro B, et al. Predicting slaughter weight in pigs with regression tree ensembles. In: APPIS. Amsterdam: Elsevier. 2018. p. 1–9.

[34] Murauer B, Specht G. Detecting music genre using extreme gradient boosting. In: Companion proceedings of the the web conference 2018. ACM. 2018. p. 1923–7.

[35] Li A, Ge M, Zhang Y, et al. Predicting long noncoding RNA and protein interactions using heterogeneous network model. BioMed Res Int. 2015 (2015). 10.1155/2015/671950 PMC4709602 26839884

[36] Zheng X, Wang Y, Tian K (2017). Fusing multiple protein–protein similarity networks to effectively predict lncRNA–protein interactions. BMC Bioinform.

[37] Zhang W, Qu Q, Zhang Y (2018). The linear neighborhood propagation method for predicting long non-coding RNA–protein interactions. Neurocomputing.

[38] Consortium U. Uniprot: a worldwide hub of protein knowledge. Nucleic Acids Res. 2019;47(D1):D506–15. 10.1093/nar/gky1049 PMC6323992 30395287

[39] Yuan J, Wu W, Xie C-Z, et al. Npinter v2.0: an updated database of ncRNA interactions. Nucleic Acids Res. 2014;42(D1): D104–D108. 10.1093/nar/gkt1057 PMC3965026 24217916

[40] Xie C-Z, Yuan J, Li H (2014). Noncodev4: exploring the world of long non-coding RNA genes. Nucleic Acids Res.

[41] Pandurangan AP, Stahlhacke J, Oates ME, et al. The superfamily 2.0 database: a significant proteome update and a new webserver. Nucleic Acids Res. 2019;47(D1): D490–4. 10.1093/nar/gky1130 PMC6324026 30445555

[42] Bai Y, Dai X, Ye T (2019). PlncRNADB: a repository of plant lncRNAs and lncRNA-RBP protein interactions. Curr Bioinform.

[43] Zhou L, Duan Q, Tian X (2021). LPI-HyADBS: a hybrid framework for lncRNA–protein interaction prediction integrating feature selection and classification. BMC Bioinform.

[44] Zhou Y-K, Hu J, Shen Z-A, Zhang W-Y, Du P-F (2020). LPI-SKF: predicting lncRNA–protein interactions using similarity kernel fusions. Fronti Genet.

[45] Liu H, Ren G, Hu H, Zhang L, Ai H, Zhang W, Zhao Q (2017). LPI-NRLMF: lncRNA–protein interaction prediction by neighborhood regularized logistic matrix factorization. Oncotarget.

[46] Hu H, Zhu C, Ai H, Zhang L, Zhao J, Zhao Q, Liu H (2017). LPI-ETSLP: lncRNA–protein interaction prediction using eigenvalue transformation-based semi-supervised link prediction. Mol bioSyst.

